# Assessment of risk factors and ultrasonographic characteristics for the differentiation between malignant and benign thyroid nodules in Beni-Suef governorate, Egypt

**DOI:** 10.1186/s12902-025-02038-4

**Published:** 2025-09-09

**Authors:** Ahmed Moheyeldien Hamed, Khaled Elsayed Elhadidy, Mahmoud Farid Kamel, Ahmed Sayed Abd El Basset, Ahmed Saad Ahmed, Saeed M. Shaaban, Nadia Ahmed Abd El-Moeze, Hazem Samy Matar

**Affiliations:** 1 Internal Medicine Department, Faculty of Medicine, Beni-Suef University, Beni-Suef City, 62514 Egypt; 2 Radiology Department, Faculty of Medicine, Beni-Suef University, Beni- Suef City, Egypt; 3 General Surgery Department, Faculty of Medicine, Beni-Suef University, Beni-Suef City, Egypt; 4 Clinical Oncology Department, Faculty of Medicine, Beni-Suef University, Beni-Suef City, Egypt; 5 Pathology Department, Faculty of Medicine, Beni-Suef University, Beni- Suef City, Egypt

**Keywords:** Thyroid nodules, TIRADS, Histopathology

## Abstract

**Background:**

Thyroid nodules (TNs) are frequent and often benign. Accurately differentiating between benign and malignant nodules is crucial for proper management. This research aims to use ultrasonography to examine TNs and identify possible risk factors in order to improve patient outcomes and diagnostic accuracy.

**Methods:**

The study included 128 euthyroid participants who underwent thyroidectomy, splitted into two groups (benign and malignant) regarding the histopathological outcomes. Data on age, sex, family history of thyroid cancer and radiation exposure were collected. Ultrasound (US) was used to assess nodule number, size, vascularity and TIRAD scores. US Lymph node status was also evaluated. Statistical analysis compared benign and malignant nodules.

**Results:**

No significant differences were found between benign and malignant groups regarding age, sex, family history, and radiation exposure. Significant differences were observed in nodule size (*p* < 0.05), echogenicity (*p* < 0.001), and margins (*p* < 0.05), with larger, hyper/isoechoic, and smooth-margined nodules more common in the benign group. TIRAD scores (*p* < 0.001) and lymph node status (*p* < 0.001) also differed significantly, with benign cases showing TR3 scores and non-suspicious lymph nodes, while malignant cases had more TR4 scores and suspicious lymph nodes. Additionally, malignant nodules were significantly more hypoechoic (*p* < 0.001). Most benign cases were nodular colloid hyperplasia, followed by follicular adenoma and thyroiditis. Most malignant cases were Papillary thyroid carcinoma (PTC) and follicular thyroid carcinoma (FTC). PTC was associated with younger age (*p* = 0.006), smaller nodule size (*p* = 0.04), and hypoechoic nodules (*p* = 0.04).

**Conclusion:**

Sex, age, family history of thyroid cancer, and radiation exposure history did not significantly vary between groups with benign and malignant thyroid tumors, according to the research. Higher TIRAD scores and hypoechoic nodules were more common in malignant nodules. Benign nodules had smoother margins, were bigger, and were more likely to be hyper/isoechoic.

## Background

The population being assessed and the screening method used have the most effects on the occurrence of TNs, an extremely prevalent endocrine disorder. The prevalence likely by palpation only is 4–7%, however US has shown that the prevalence of TNs in the adults may reach 20–76%, mainly with the adoption of high-resolution US procedures in recent years [1].

Usually, 2–6% of individuals have benign TNs discovered during physical exams, 19–68% on ultrasounds, and 8–65% following autopsy. Although most are benign, the chance of getting cancer may vary from 7 to 15% contingent upon age, sex, radiation exposure, and family history [2].

Better access to healthcare in general and the expanding use of diagnostic imaging and medical monitoring are the main causes of the increasing frequency of thyroid cancer diagnosis. Even while epidemiological studies show a minor but genuine rise in the prevalence of malignant thyroid lesions, all of these circumstances promote the diagnosis of small, subclinical TNs and small papillary thyroid tumors [3].

More than 95% of thyroid malignancy exhibit several neoplastic phenotypes, resembling anaplastic thyroid cancer, FTC, and PTC. PTC account for about 85–90% of the thyroid malignancies, according to different populations observed. Medullary thyroid malignancies are rare tumors of thyroid cells that release calcitonin, accounting for 5–10% of all thyroid cancers [4].

Ultrasound is the primary imaging method for evaluating TNs and is often used as a guide for less invasive procedures. With new tools and software, US technology is always evolving and might aid medical professionals in treating TNs [5].

This study included pathologically established benign and malignant TNs from patients who underwent total or partial thyroidectomy for management of thyroid nodule(s) to compare the ultrasonic characteristics of different types of nodules and assess other potential risk factors in Beni-Suef Governorate. Therefore, we could identify patients with high risk to have malignant lesion based on simple non-invasive procedure.

## Methods

This study was a prospective observational study performed at Beni-Suef University Hospital starting in May 2023 till Oct 2024. The study was performed on 128 patients operated for thyroid nodule(s) either by partial or total thyroidectomy. Following histopathological examination, patients were allocated to either group (A) including 64 patients with benign TNs and group (B) including 64 patients with malignant thyroid nodule(s). Patients with thyroid dysfunctions, either hyperthyroidism or hypothyroidism were excluded from the study.

Every patient underwent complete history taking with special focus on age, gender, family history and radiation exposure of thyroid cancer. Thyroid function tests were evaluated before the operation, then all patients were scheduled for preoperative thyroid ultrasonography.

### Thyroid ultrasonography

Thyroid US was Applied at the Radiology Department, Beni-Suef University Hospital using (Ge logiq S6 10 L probe) Model. Data obtained from the US assessment included the number of TNs Present (Solitary Thyroid Nodule (STN) or Multinodular (MNG)). Description of Thyroid Nodule(s) included the size of the nodule and in case of MNG the most doubtful or dominant one was described.

**Fig. 1:**
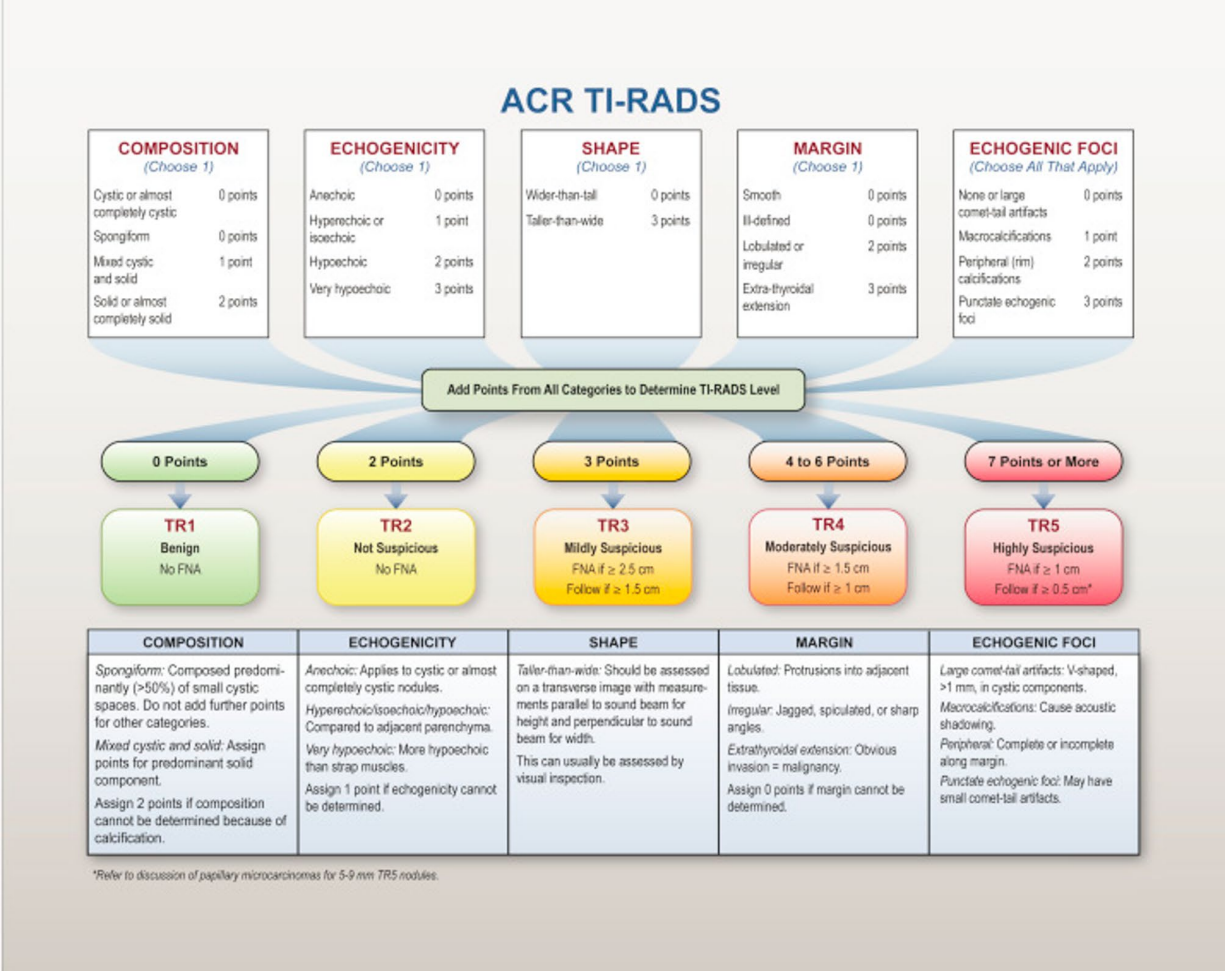
Five categories of the ACR thyroid imaging, reporting and data system (TI-RADS) [6]

Then we applied ACR- TIRADS Thyroid nodule(s) status (composition, shape, margin, echogenicity and echogenic foci) and Calculation of the Score according to Tessler et al. as shown in (Fig. 1) [6]. Also, the vascularity of Thyroid Nodule(s) was described as Non or hypo vascular, peripheral vascularity, central vascularity or peripheral & central vascularity. Moreover, the LNs were evaluated either suspicious or non-suspicious.

### Ethical considerations

Informed consent was taken from all the patients before the study and after ethical committee approval in Beni-Suef, Faculty of Medicine. Approval No:** (**FMBSUREC/02052023/Hamed).

### Statistical analysis

For this data analysis, we consulted SPSS Inc.‘s (Chicago, IL, USA) Statistical Package of Social Science (SPSS) 22 for Windows 7. Standard deviations are a measure of the dispersion of quantitative parametric data, arithmetic means are a way to measure central tendency, and simple descriptive analysis is expressed as numbers and percentages of qualitative data. For non-parametric quantitative data, the median and range. Regarding quantitative parametric data: Separate samples Two separate groups’ quantitative measurements were compared using the t test. When comparing quantitative measurements between more than two independent groups of quantitative data, the one-way ANOVA test is utilized. Regarding qualitative data: When comparing two or more qualitative groups, the chi square test is utilized. Statistical significance was determined by a P-value lower than 0.05.

## Results

This study includes 128 Euthyroid patients divided into two equal groups (64 Benign and 64 Malignant) depending on histopathological results after thyroidectomy for management of Thyroid Nodule(s). Among benign cases 65.6% of them were nodular colloid hyperplasia, 25% were follicular adenoma, and 9.4% were thyroiditis by histopathology analysis. Among the malignant group, 79.7% of them were PTC, and 7.8% were FTC, 3.1% were Poorly Differentiated Thyroid Carcinoma, 3.1% were Hurthle cell Carcinoma, 3.1% were Lymphoma, 1.6% were Medullary Thyroid Carcinoma, and 1.6% were Anaplastic Thyroid Carcinoma.

### Demographic data of the studied patients (Table 1)

**Table 1:** Comparisons of demographic characters in different postoperative thyroid histopathology results

Variables	Benign (*N* = 64)		Malignant (*N* = 64)		*P*-value	Sig.	
	Mean	SD	Mean	SD			
Age (years)	44.4	13.7	44.9	12.6	0.84		NS
Sex	No.	%	No.	%			
Male	7	10.9%	8	12.5%	0.99	NS	
Female	57	89.1%	56	87.5%			

The mean age of patients with benign lesions was (44.4 ± 13.7 years) and most of them were females (89.1%). Similarly, the mean age of patients with malignant lesions was (44.9 ± 12.6 years) and most of them were females (87.5%). Statistical analysis did not reveal any discernible difference between the groups of thyroid tumors classified as benign or malignant. Concerning their age (P-value 0.84) and sex (P-value 0.99).

### Medical history of the studied patients (Table 2)

**Table 2:** Comparisons of medical history in different postoperative thyroid histopathology results

Variables	Benign (*N* = 64)		Malignant (*N* = 64)		*P*-value	Sig.
	Mean	SD	Mean	SD		
TSH level	1.87	0.67	1.79	0.85	0.58	NS
Family history of thyroid cancer					0.49	NS
Negative	64	100%	62	96.9%		
Positive	0	0%	2	3.1%		
Exposure to radiation					1	NS
No	63	98.4%	64	100%		
Yes	1	1.6%	0	0%		

TSH levels were within normal in both group with average (1.87 ± 0.67) in patients with benign lesions and (1.79 ± 0.85) in patients with malignant lesions (P-value 0.58). Only 2 patients (3.1%) in malignant lesions group showed family history of thyroid cancer and 1 patient (1.6%) was exposed to radiation in benign lesions group. There was no statistically significant difference between benign and malignant thyroid tumor groups as regards family history of thyroid Cancer (P-value 0.49), and history of radiation exposure (P-value 1).

### Ultrasound features of the studied patients and the associated TIRADs score (Table 3)

**Table 3:** Comparisons of US features in different postoperative thyroid histopathology results

Variables	Benign (*N* = 64)		Malignant (*N* = 64)		P-value	Sig.
	No.	%	No.	%		
Number of TNs					0.56	NS
Solitary	16	25%	20	31.3%		
Multinodular	48	75%	44	68.8%		
Nodule size					**0.002**	**HS**
Less than or equal 1 cm	2	3.1%	10	15.6%		
1.1 to 1.9 cm	7	10.9%	20	31.3%		
2 to 2.9 cm	16	25%	9	14.1%		
3 to 3.9 cm	20	31.3%	10	15.6%		
More than or equal 4 cm	19	29.7%	15	23.4%		
Nodule vascularity					0.68	NS
Non or hypo vascular	26	40.6%	28	43.7%		
Peripheral vascularity	13	20.3%	9	14.1%		
Central vascularity	11	17.2%	9	14.1%		
Peripheral & Central	14	21.9%	18	28.1%		
Nodule shape					0.12	NS
Wider than taller	63	98.4%	58	90.6%		
Taller than wider	1	1.6%	6	9.4%		
Nodule margin					**< 0.001**	**HS**
Smooth/Ill defined	64	100%	48	75%		
Lobulated/Irregular	0	0%	14	21.9%		
Extra-thyroid extension	0	0%	2	3.1%		
Nodule composition					0.28	NS
Cystic/spongiform	1	1.6%	0	0%		
Mixed solid and cystic	8	12.5%	4	6.3%		
Solid	55	85.9%	60	93.7%		
Nodule echogenicity					**< 0.001**	**HS**
Anechoic	0	0%	1	1.6%		
Hyperechoic or isoechoic	56	87.5%	23	35.9%		
Hypoechoic	8	12.5%	40	62.5%		
Nodule echogenic foci					0.26	NS
None or large comet-tail artifact	48	75%	43	67.2%		
Macro calcifications	10	15.6%	7	10.9%		
Peripheral/rim calcifications	1	1.6%	2	3.1%		
Punctate echogenic foci	5	7.8%	12	18.8%		
TIRAD score					< 0.001	HS
TRII: No suspicion	5	7.8%	0	0%		
TRIII: Mild suspicion	38	59.4%	14	21.9%		
TRIV: Moderate suspicion	19	29.7%	35	54.7%		
TRV: High suspicion	2	3.1%	15	23.4%		

Most patients with benign and malignant lesions had multinodular goiter (75% and 68.8%, respectively) with no significant variations between groups (P-value 0.56). However, the size of the nodules varied significantly by US with a higher percentage of larger size among benign tumor group (P-value 0.002). Also, there was a statistically significant difference in nodular echogenicity by US with higher percentage of hyper/isoechoic among benign tumor group but higher percentage of hypoechoic among malignant group (P-value < 0.001). In addition, there was a statistically significant difference in nodular margin with higher percentage of smooth margin among benign tumor group (P-value < 0.001).

Statistical analysis of nodular vascularity, however, failed to distinguish between benign and malignant thyroid tumor groups. (P-value 0.68), nodular shape (P-value 0.12), nodule composition (P-value 0.28), and nodule echogenic foci (P-value 0.26).

There was a statistically significant difference in TIRAD score with higher percentage of TR3 among benign tumor group (59.4%) but higher percentage of TR4 among malignant group (54.7%) (P-value < 0.001).

### LNs status by US among the studied patients (Table 4)

**Table 4:** Comparisons of TIRAD score and LNs status by US in different postoperative thyroid histopathology results

Items	Benign (*N* = 64)		Malignant (*N* = 64)		P-value	Sig.
	No.	%	No.	%		
Lymph Nodes status					< 0.001	HS
Not suspicious	64	100%	52	81.3%		
Suspicious	0	0%	12	18.8%		

All LNs in the benign group were non-suspicious (100%), while in malignant group LNs were suspicious in (18.8%) with significant differences between both groups (P-value < 0.001).

### Comparison between US nodular characteristics in different benign and malignant lesions (Tables 5 and 6)

**Table 5:** Comparisons of US nodular characters in different postoperative thyroid benign histopathology results

Variables	Benign (*N* = 64)			*P*-value
	Follicular adenoma	Thyroiditis	Nodular colloid hyperplasia	
Age (years)	38.5 ± 12.2	50.8 ± 6.1	45.8 ± 14.4	0.09
Sex				0.66
Male	2(12.5%)	0(0%)	5(11.9%)	
Female	14(85.5%)	6(100%)	37(88.1%)	
Number of TNs				< 0.001*
Solitary	11(68.8%)	0(0%)	5(11.9%)	
Multinodular	5(31.30%)	6(100%)	37(88.1%)	
Nodule size				0.001*
Less than or equal 1 cm	0(0%)	2(33.3%)	0(0%)	
1.1 to 1.9 cm	1(6.3%)	0(0%)	6(14.3%)	
2 to 2.9 cm	5(31.3%)	1(16.7%)	10(23.8%)	
3 to 3.9 cm	2(12.5%)	2(33.3%)	16(38.1%)	
More than or equal 4 cm	8(50%)	1(16.7%)	10(23.8%)	
Nodule shape				0.77
Wider than tall	16(100%)	6(100%)	41(97.6%)	
Taller than wide	0(0%)	0(0%)	1(2.4%)	
Nodule composition				0.96
Cystic/spongiform	0(0%)	0(0%)	1(2.4%)	
Mixed solid and cystic	2(12.5%)	1(16.7%)	5(11.9%)	
Solid	14(87.5%)	5(83.3%)	36(85.7%)	
Nodule echogenicity				0.94
Hyper- or isoechoic	14(87.5%)	5(83.3%)	37(88.1%)	
Hypoechoic	2(12.5%)	1(16.7%)	5(11.9%)	
Nodule echogenic foci				0.54
None or large comet-tail artifact	13(81.3%)	5(83.3%)	30(71.4%)	
Macro calcifications	1(6.3%)	0(0%)	9(21.4%)	
Peripheral/rim calcifications	0(0%)	0(0%)	1(2.4%)	
Punctate echogenic foci	2(12.5%)	1(16.7%)	2(4.8%)	
Nodule vascularity				0.79
Non or hypo vascular	5(31.3%)	3(50%)	18(42.9%)	
Peripheral vascularity	3(18.8%)	2(33.3%)	8(19%)	
Central vascularity	3(18.8%)	0(0%)	8(19%)	
Peripheral & Central	5(31.3%)	1(16.7%)	8(19%)	

In benign tumor group there was a statistically significant higher percentage of solitary nodules and large size ≥ 4 cm among Follicular adenoma tumor group but higher percentage of multinodular in thyroiditis and nodular colloid hyperplasia with size between 3 and 3.9 cm (P-value < 0.001). On the other hand, there was no statistically significant difference between benign thyroid tumor types as regards age (P-value 0.09), sex (P-value 0.66), nodular shape (P-value 0.77), composition (P-value 0.96), echogenicity (P-value 0.94), and vascularity (p-value 0.79).

**Table 6:** Comparisons of US nodular characters in different postoperative thyroid benign histopathology results

Variables	Malignant (*N* = 64)		*P*-value
	Papillary carcinoma	Other	
Age (years)	42.8 ± 11.9	53.3 ± 12.5	0.006*
Sex			0.66
Male	6(11.8%)	2(15.4%)	
Female	45(88.2%)	11(84.6%)	
Number of TNs			0.99
Solitary	16(31.4%)	4(30.8%)	
Multinodular	35(68.6%)	9(69.2%)	
Nodule size			0.04*
Less than or equal 1 cm	9(17.6%)	1(7.7%)	
1.1 to 1.9 cm	18(35.3%)	2(15.4%)	
2 to 2.9 cm	9(17.6%)	0(0%)	
3 to 3.9 cm	6(11.8%)	4(30.8%)	
More than or equal 4 cm	9(17.6%)	6(46.2%)	
Nodule shape			0.59
Wider than tall	47(92.2%)	11(84.6%)	
Taller than wide	4(7.8%)	2(15.4%)	
Nodule margin			0.35
Smooth/Ill-defined	40(78.4%)	8(61.5%)	
Nobulated/Irregular	10(19.6%)	4(30.8%)	
Extra-thyroidal extension	1(2%)	1(7.7%)	
Nodule composition			0.99
Mixed cystic and solid	3(5.9%)	1(7.7%)	
Solid	48(94.1%)	12(92.3%)	
Nodule echogenicity			0.04*
Anechoic	0(0%)	1(7.7%)	
Hyper- or isoechoic	21(41.2%)	2(15.4%)	
Hypoechoic	30(58.8%)	10(76.9%)	
Nodule echogenic foci			0.15
None/large comet-tail artifact	31(60.8%)	12(92.3%)	
Macro-calcifications	6(11.8%)	1(7.7%)	
Peripheral/rim calcifications	2(3.9%)	0(0%)	
Punctate echogenic foci	12(23.5%)	0(0%)	
Nodule vascularity			0.40
Non or hypo vascular	23(45.1%)	5(38.5%)	
Peripheral vascularity	8(15.7%)	1(7.7%)	
Central vascularity	8(15.7%)	1(7.7%)	
Peripheral & Central	12(23.5%)	6(46.2%)	
Lymph Nodes status	(%)	(%)	0.24
Not suspicious	43(84.3%)	9(69.2%)	
Suspicious	8(15.7%)	4(30.8%)	

In malignant tumor group there was a statistically significant younger age (P-value 0.006), small size between 1.1 and 1.9 cm (P-value 0.04), and hypoechoic nodules (P-value 0.04) among cases with PTC. On the other hand, there was no statistical significant difference between benign thyroid tumor types as regards sex (P-value 0.66), nodular number (P-value 0.99), shape (P-value 0.59), margin (P-value 0.35), composition (P-value 0.99), and vascularity (P-value 0.4).

## Discussion

Researchers and physicians have focused a lot of interest on the study of TNs. There is a greater risk of malignancy in some TNs. Thyroid cancer may be successfully prevented and major repercussions can be avoided with early thyroid nodule identification and treatment. How to differentiate malignant nodules from a large number of TNs and how to separate benign and malignant nodules from patient clinical data are significant and challenging problems in the clinic [7].

There are many clinical techniques, such as nuclear scanning, computed tomography scans, and US, to differentiate between benign and malignant TNs. Due to its numerous benefits, including safety, affordability, ease of use, repeatability, and lack of radioactivity, conventional US examination has been utilized extensively in clinical practice [8].

Therefore, this study aimed to compare the ultrasonic features of various thyroid nodule types, such as number, size, vascularity, composition, echogenicity, shape, margin, echogenic foci, and lymph node assessment, as well as other potential risk factors (age, gender, radiation exposure, and family history of thyroid cancer). This study included pathologically confirmed benign or malignant TNs.

This study comprised 128 euthyroid patients, who were categorized into two equal groups 64 individuals each (benign and malignant) based on histopathological outcomes following thyroidectomy for the management of thyroid nodule(s).

Regarding age, sex, family history of thyroid cancer, and history of radiation exposure, our results showed that there were no statistically significant differences between the groups with benign and malignant thyroid tumors.

In line with our findings, Nixon et al. came to the conclusion that, despite its rarity, familial non-medullary thyroid carcinoma is clinically diagnosable. It is still up to debate how this syndrome affects prognostic variables and how it then affects management options. Treatment strategies for people with low risk differentiated thyroid cancer should be based on risk evaluations that account for familial non-medullary thyroid cancer [9].

Furthermore, Byun et al. showed that the incidence of thyroid cancer did not change with the development of thyroid cancer in fathers or siblings. Mothers of participants with an early age of onset (group aged < 50 years, *p* = 0.007), however, had a greater incidence. This study emphasized the link between a family history of thyroid cancer and the incidence of thyroid cancer.

There was no statistically significant relationship between radiation exposure and thyroid cancer incidence in our investigation. This lack of association may be attributed to the absence of significant radiation activities in our study area. Furthermore, we did not conduct a cohort study to monitor individuals with a history of radiation exposure over time [10].

A statistically significant variation was observed in nodular size by US with higher percentage of larger size among benign tumor group with p-value < 0.05. However, no statistically significant differences were detected between the benign and malignant thyroid tumor groups regarding the number of TNs and nodular vascularity.

There was a noticeable disparity that met statistical significance in nodular echogenicity as assessed by US, with a greater proportion of hyper/isoechoic nodules in the benign tumor group and a higher percentage of hypoechoic nodules in the malignant group (p-value < 0 0.001). Additionally, there was a significant difference in nodular margins, where the benign tumor group exhibited a higher percentage of smooth margins (p-value < 0 0.05). However, no statistically significant differences were found between the benign and malignant thyroid tumor groups concerning the composition of TNs, nodular shape, or the presence of echogenic foci, with p-value > 0.05.Furthermore, a significant difference in TIRAD scores was noted, with a higher percentage of TR3 scores in the benign tumor group compared to a greater percentage of TR4 scores in the malignant group (p-value < 0.001).

Lastly, there was a statistically significant difference in lymph node status as observed by US, showing a higher percentage of non-suspicious findings in the benign tumor group, while the malignant group had a greater proportion of suspicious findings (p-value < 0.001).

We found similar findings in a meta-analysis that comprised seven trials conducted by Hammad et al. with 10,817 TNs, 2,206 (20.4%) of which were malignant. The analysis showed that nodules sized from 3 cm to 5.9 cm had a 26% higher risk of malignancy than those less than 3 cm (OR 1.26; 95% CI: 1.13–1.39) after controlling for factors such patient age and gender. Curiously, nodules 6 cm or bigger had a 16% lower risk of cancer than nodules less than 3 cm (OR 0.84; 95% CI: 0.73–0.98) [11]. Out of 4,154 thyroid nodules evaluated in 14 prospective studies, 1,419 (or 34% of the total) were found to be malignant, according to Khadra et al., who reviewed 89 publications in total. According to the study, 33% of malignant nodules lacked vascular perfusion [12].

We found that the number of TNs, STN vs. MNG showed a different trend from the analysis by Rehman et al. which included 22 studies from 1992 to 2018 and a cohort of 50,321 patients. Of the patients in this meta-analysis, 55.37% had MNG and 44.2% had STN. The findings showed that, in comparison to STN, MNG was linked to a noticeably decreased risk of thyroid cancer [13].

Lee et al. conducted a retrospective multicenter validation study where 5,601 TNs with final diagnosis were evaluated from 26 different institutions. Nodules were categorized according to echotexture (homogeneous vs. heterogeneous) and hypoechogenicity (mild, moderate, or marked). The risk of malignancy was determined based on suspicious characteristics and composition. Heterogeneous hypoechoic nodules were shown to have a considerably higher probability of malignancy than heterogeneous isoechoic nodules (*P* < 0.017) [14].

Shen et al. assessed various risk-stratification methods for identifying TNs that are benign or malignant. The results corroborated our findings, showing that the malignant nodules had a significantly higher likelihood of being solid or mostly solid, hypoechogenic or very hypoechogenic, taller-than-wide, having lobulated or irregular margins, extrathyroidal extension, microcalcifications, and lymph node metastases than the benign nodules (*P* < 0.05 for all) [15].

Januś et al. examined the histological and ultrasonography evaluation of benign, borderline, and malignant thyroid cancers. Similar to our results, they showed that specific characteristics, such as microcalcifications, noticeable hypoechogenicity, lobulated or irregular ill-defined borders, and extrathyroidal invasion, were only seen in malignant nodules [16].

Age (*p* = 0.031), nodule size (*p* < 0.001), heterogeneous enhancement (*p* < 0.001), hypo-enhancement (*p* = 0.001), unclear margin (*p* = 0.007), inner peak (*p* < 0.001), and outside sharpness (*p* < 0.001) were all significantly different between benign and malignant nodules, according to Fan et al. [17].

This classification system is based on the idea that the more suspicious US features there are and the less benign findings there are, the higher the risk of cancer. Research by Horvath et al. comprised 502 nodules from 210 patients validated the TI-RADS categorization. According to the study, the TI-RADS sensitivity for malignancy was 99.6% (95% CI: 98.9–100.0%), the specificity was 74.35% (95% CI: 68.70–80.00%), the PPV was 82.1% (95% CI: 78.0–86.3%), and the NPV was 99.4% (95% CI: 98.3–100.0%). In TI-RADS 2, the estimated probability of malignancy was 0%; in TI-RADS 3, it was 3.4%; in TI-RADS 4, it was 10–80%; and in TI-RADS 5, it was 87% [18].

In their study, Luo et al. showed that using SWE in conjunction with ACR TI-RADS categorization helped enhance thyroid nodule diagnosis. The authors used share wave elastography to find nodules that met the criteria for ACR TI-RADS 4 and had a high chance of being benign. They come to the conclusion that this process might reduce the number of needless biopsies of benign TNS and aid in the identification and selection of benign nodules [19].

On the other hand, a risk categorization method for TNs was created by Liang et al. using five ultrasonography findings. After analyzing 1,236 nodules from 999 patients, the study divided the patients into three different risk groups. The Korean Thyroid Imaging Reporting and Data System (K-TIRADS) and the American College of Radiology Thyroid Imaging Reporting and Data System (ACR TIRADS) were both compared with the updated Thyroid Imaging Reporting and Data System (R-TIRADS), which showed better diagnostic performance and higher sensitivity. Due to this development, a considerable decrease in needless biopsies occurred [20].

In the benign cases, histopathological examination showed that 65.6% were nodular colloid hyperplasia, 25% were follicular adenoma, and 9.4% were thyroiditis. In malignant cases, 79.7% were identified as PTC, and 7.8% were FTC. Within the benign tumor group, a significantly higher percentage of solitary nodules and larger size (≥ 4 cm) was observed in the follicular adenoma subgroup, whereas multinodular presentations were more frequent in cases of thyroiditis and nodular colloid hyperplasia, with nodule sizes ranging from 3 to 3.9 cm (*p* < 0.001). There was no statistically significant difference among benign thyroid tumor types with respect to age, sex, nodule shape, composition, echogenicity, and vascularity (*p* > 0.05).

In the malignant tumor group, cases of PTC were associated with significantly younger age, smaller nodule size (1.1–1.9 cm), and hypoechoic nodules, with p-values of 0.006, 0.04, and 0.04, respectively. Regarding sex, nodule number, form, margin, composition, and vascularity, however, there were no statistically significant variations among benign thyroid tumor types. (*p* > 0.05).

Our findings align with LeClair et al. cohort study, which reported that the current incidence ratio of small (≤ 2 cm) PTC in women to men is 4.39:1 [21].

Our study highlights the significance of the risk factors and US features for differentiation between benign and malignant thyroid lesions prior to any intervention or further invasive investigations.

## Conclusions

Sex, age, family history of thyroid cancer, and radiation exposure history did not significantly vary between groups with benign and malignant thyroid tumors, according to the research. Higher TIRAD scores and hypoechoic nodules were more common in malignant nodules. Benign nodules had smoother margins, were bigger, and were more likely to be hyper/isoechoic.

## Data Availability

Please contact the corresponding author for any data required.
